# Antimicrobial-resistant Gram-negative colonization in infants from a neonatal intensive care unit in Thailand

**DOI:** 10.1016/j.jhin.2019.04.004

**Published:** 2019-10

**Authors:** T. Roberts, D. Limmathurotsakul, P. Turner, N.P.J. Day, W.P. Vandepitte, B.S. Cooper

**Affiliations:** a Mahidol-Oxford Tropical Medicine Research Unit, Faculty of Tropical Medicine, Mahidol University, Bangkok, Thailand; b Lao-Oxford-Mahosot Hospital-Wellcome Trust Research Unit, Microbiology Laboratory, Mahosot Hospital, Vientiane, Lao Democratic People's Republic; c Cambodia Oxford Medical Research Unit, Angkor Hospital for Children, Siem Reap, Cambodia; d Department of Paediatrics, College of Medicine, Rangsit University, Bangkok, Thailand; e Division of Infectious Diseases, Department of Paediatrics, Queen Sirikit National Institute of Child Health, Bangkok, Thailand; f Centre for Tropical Medicine and Global Health, Nuffield Department of Medicine, University of Oxford, Oxford, UK

**Keywords:** Extended-spectrum beta-lactamase, Carbapenem resistance, Antimicrobial resistance, Neonate, Colonization

## Abstract

Antimicrobial-resistant Gram-negative bacteria are a major cause of morbidity and mortality in hospitalized neonates in South and South-East Asia. This study aimed to determine the dynamics of colonization with antimicrobial-resistant Gram-negative bacteria amongst patients in a neonatal intensive care unit (NICU) in Thailand. From 97 enrolled patients, 52% were colonized by an extended-spectrum β-lactamase (ESBL) organism at some point during their stay and 64% were colonized by a carbapenem-resistant organism. Rapid acquisition of ESBL-positive and carbapenem-resistant organisms was found. Once colonized with an antibiotic-resistant organism, patients remained colonized for the remainder of their NICU stay.

## Introduction

Antibiotic-resistant Gram-negative bacteria including Enterobacteriaceae, *Acinetobacter baumannii* and *Pseudomonas aeruginosa* have been identified by the World Health Organization (WHO) as critical priorities for new treatment options [1]. In South-East Asia, these pathogens account for a high and increasing disease burden, and the risk of emergence and spread of antibiotic resistance in the WHO South East Asia region is thought to be among the highest of all the WHO regions [2], [3]. In Thailand, over 80% of the estimated 19,000 annual excess deaths due to antibiotic-resistant bacteria result from Gram-negative pathogens [4]. There is an immediate need for effective infection control interventions to reduce this disease burden. However, the population dynamic processes driving these increases and sustaining the high level of endemicity are poorly understood. Such understanding depends on knowledge of colonization dynamics of antibiotic-resistant bacteria in hospital settings. This is important because asymptomatic colonization typically precedes clinical infection, colonized patients act as a reservoir for the spread of resistant organisms throughout the hospital, and infection control interventions such as hand hygiene work primarily by reducing the burden of such asymptomatic colonization [2].

Patients in neonatal intensive care units (NICUs) are particularly vulnerable to life-threatening infections with these organisms. Previous work has highlighted the importance of asymptomatic carriage for understanding the transmission dynamics of methicillin-resistant *Staphylococcus aureus* (a Gram-positive pathogen) in the NICU setting in Thailand [5]. However, longitudinal carriage studies for antibiotic-resistant Gram-negative pathogens are scarce and, to the authors' knowledge, have not been performed previously in Thailand. To address this knowledge gap, this study aimed to characterize the dynamics of infant colonization with antibiotic-resistant Gram-negative bacteria in patients in an NICU in Bangkok, Thailand.

## Materials and methods

### Study site

This study was conducted at a large referral centre in Bangkok, Thailand. The centre has 426 beds for outpatient, inpatient, intensive care and surgical departments, with approximately 15,400 inpatients and 401,000 outpatients annually. The NICU has eight beds. There is no maternity unit at the centre so all patients are born off-site.

### Study subjects

This longitudinal study included all patients admitted to the NICU between February and September 2015. Written informed consent was given for all subjects enrolled in the study by their parents or legal guardians. Ethical approval was obtained from Oxford Tropical Research Ethics Committee (Reference 7–15) and from the local hospital ethics committee.

### Study samples

After enrolment in the study, patients underwent twice-weekly colonization screening, comprising rectal swabs (all patients) and throat swabs (only if intubated/ventilated). A stool sample was collected weekly from each patient where possible. Samples underwent full microbiological testing using standard microbiological techniques and API 20E/20NE (bioMérieux, Marcy L'Etoile, France). Comprehensive antimicrobial resistance profiles were determined following current US Clinical and Laboratory Standards Institute guidelines (M100S, 26^th^ edition, January 2016) using disk diffusion on Mueller-Hinton agar (Oxoid, Basingstoke, UK). A full description of microbiological testing is given in Supplementary Material I (see online).

### Statistical analysis

Data were analysed using Stata Version 14 (StataCorp, College Station, TX, USA). Supplementary Material II (see online) gives a full description of statistical analysis.

## Results

### Study population

In total, 97 patients were enrolled in the study (49 male, 48 female). Ninety-two patients were born at another hospital, one patient was born in a healthcare centre, one patient was born at home, and the birth place was not known for the remaining three patients. Sixty-six patients (68%) were premature. Sixty-nine patients were 0 days old on admission, 16 patients were aged 1–3 days, five patients were aged 3–7 days, and nine patients were aged between 8 and 28 days. The median duration of admission in the NICU was 7 days (range 1–98 days). Two patients were re-admitted to the NICU during the study, and there were 12 deaths (12%) during NICU admission. The mean discharge weight was 1.93 kg (weight at enrolment was unknown).

### Infant colonization

Medians of two (range one to 18) rectal swabs, two (range one to 18) tracheal swabs and one (range one to five) stool sample were collected per patient. From a total of 660 samples (341 rectal swabs, 243 tracheal swabs and 76 stool samples), 890 organisms with some form of antimicrobial resistance were isolated; 165 samples had no organism isolated. There were 322 *Klebsiella pneumoniae*, 223 *Pseudomonas aeruginosa*, 190 *Acinetobacter baumannii*, 115 *Enterobacter* spp*.*, 38 *Escherichia coli* and two *Acinetobacter lwoffii* [results and site of colonization are summarized in Table A (see online supplementary material)]. Overall, 69 of 97 (71%) patients were colonized by at least one antibiotic-resistant Gram-negative organism, and all 69 patients were colonized with at least one third-generation cephalosporin**-**resistant (3GC-R) organism. *A. baumannii* was isolated from 55 (57%) patients and *K. pneumoniae* was isolated from 50 (52%) patients. Fifty patients had an extended-spectrum beta-lactamase (ESBL)-positive organism isolated from at least one sample: two patients with an ESBL *E. coli* (ESBLEC) alone, 38 patients with an ESBL *K. pneumoniae* (ESBLKP) alone, and 10 patients with both ESBLEC and ESBLKP. Sixty-two patients were colonized by an imipenem-resistant (IPM-R) organism [most commonly *A. baumannii,* carried by 52 (54%) patients]. Based on initial swabs on admission to the NICU, 26 of 97 patients were colonized by an ESBL-positive organism and 37 of 97 patients were colonized by an IPM-R organism. An additional 24 patients who were ESBL-negative on their initial sample became colonized with an ESBL-positive organism within 3–75 days of admission (median 8 days), and 25 patients had an IPM-R organism isolated after their initial sample within 3–20 days of admission (median 7 days). The number of patients with antimicrobial-resistant organisms at and during admission is summarized in Table I.

**Table I:** Number of patients with an antimicrobial-resistant organism at and during admission

Organism	Initial sample positive *N* (%) 97 patients			Negative on initial sample and became colonized during admission *N* (%) 97 patients		
	3GC-R	ESBL	IPM-R	3GC-R	ESBL	IPM-R
*Escherichia coli*	7 (7)	7 (7)	0	5 (5)	5 (5)	3 (3)
*Enterobacter* spp.	15 (15.5)	-	0	19 (19.2)	-	3 (3)
*Klebsiella pneumoniae*	26 (26.8)	25 (25.7)	17 (17.5)	22 (22.7)	21 (21.6)	25 (25.2)
*Acinetobacter* spp.	25 (25.3)	-	22 (22.2)	29 (29.9)	-	30 (30.1)
*Pseudomonas aeruginosa*	11 (11.1)	-	12 (12.4)	30 (30.3)	-	32 (32.3)
Any of the above organisms	48 (49.5)	26 (26.8)	37 (38.1)	21 (21.2)	24 (24.3)	25 (25.8)

3GC-R, third-generation cephalosporin**-**resistant; ESBL, extended-spectrum beta-lactamase; IPM-R, imipenem-resistant.

**Figure 1:**
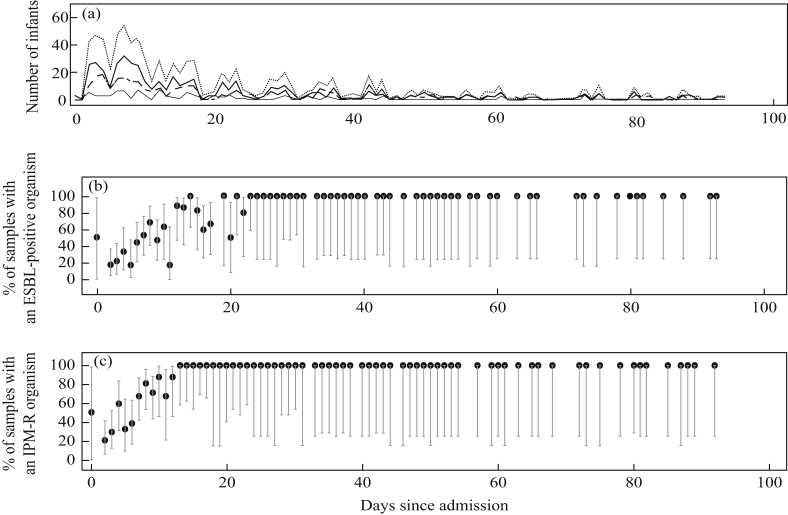
(A) Number of patients who had samples taken and the number of each different sample type taken by days since admission. Dotted line, number of samples taken; bold line, rectal swabs taken; fine line, stools taken; dashed line, tracheal swabs taken. (B) Percentage of samples that grew an extended-spectrum beta-lactamase (ESBL)-positive organism by days since admission (vertical lines show 95% confidence intervals). (C) Percentage of samples that grew an imipenem-resistant (IPM-R) organism by days since admission (vertical lines show 95% confidence intervals).

All isolates from day 13 or later of a patient's stay in the NICU from which an organism was grown were positive for an IPM-R organism. After 22 days in the NICU, all isolates from which an organism was grown were positive for an ESBL-positive organism (Figure 1). In both cases, once positive, all subsequent isolates remained positive. Isolates from samples taken over the course of a patient's stay were increasingly likely to be IPM-R or ESBL-positive with time since NICU admission, with the risk increasing steadily after 5 days. Figure A (see online supplementary material) shows the cumulative incidence function for colonization with an ESBL-positive or IPM-R organism during admission. This graph considers all patients who had an initial negative screen for the target organism.

Gentamicin resistance was also common, with 52 patients having a gentamicin-resistant organism (309 of 322 *K. pneumoniae* isolates, 183 of 190 *Acinetobacter* spp*.* isolates*,* 108 of 115 *Enterobacter* spp. isolates, 79 of 222 *P. aeruginosa* isolates and 28 of 38 *E. coli* isolates). Antibiotic resistance patterns for organisms are summarized in Table B (see online supplementary material).

From the univariable and multi-variable logistic regression model, male sex was the only factor clearly associated with ESBL-positive and IPM-R organism colonization in the initial sample with odds ratios of 3.23 (95% confidence interval (CI) 1.22–8.57) and 3.75 (95% CI 1.52–9.22), from the multi-variable regression respectively (Table C and D, see online supplementary material). In the multi-variable Cox regression, premature birth was associated with a reduced acquisition rate for both ESBL-positive and IPM-R organisms, with hazard ratios of 0.31 (95% CI 0.11–0.88) and 0.26 (95% CI 0.10–0.69), respectively (Tables E and F, see online supplementary material). From the Fine and Gray competing risks regression model, birth asphyxia was associated with an increased cumulative incidence of ESBL-positive colonization during admission (hazard ratio 2.71, 95% CI 1.25–5.87), and there was some evidence of an association between patient ventilation and increased acquisition of both ESBL-positive and IPM-R organisms although the CIs were wide (Tables G and H, see online supplementary material).

## Discussion

The prevalence rates of carriage were high on initial screening for ESBL-positive and IPM-R organisms (27% and 38%, respectively), with a similar number of patients found to be negative on their initial sample subsequently becoming positive during their NICU stay (34% and 42%, respectively). Nearly half of all patients were colonized with at least one 3GC-R organism on admission to the NICU, while a further 21% who were 3GC-R negative on admission became positive during their NICU stay. There were rapid increases in the proportion of isolates testing positive for ESBL-positive and IPM-R organisms over the course of patients' stays on the NICU; and, amongst specimens which grew an organism, 100% of isolates taken from patients who had stayed in the NICU for more than 3 weeks were positive for both. Two different processes could be driving these increases: transmission on the NICU to patients who were ESBL-/IPM-R negative on admission, or selection for initially undetectable resistant subpopulations. Future genomic and epidemiological studies are needed to quantify the relative importance of these processes. The high persistence of carriage of ESBL-positive and IPM-R organisms over timescales relevant to ward-level dynamics was highlighted by the fact that once a patient tested positive for an ESBL-positive or IPM-R organism, all subsequent isolates were positive during the NICU stay.

Two previous ESBL carriage studies from adults in Thailand identified *E. coli* as the predominant ESBL-positive organism; in contrast, in the present study population, *K. pneumoniae* accounted for the majority of ESBLs, with 50% of patients colonized at some time during their NICU stay [6], [7]. This is particularly worrying as *K. pneumoniae* is one of the most important neonatal pathogens in developing countries, with reported case fatality rates of 18–68% from bloodstream infections. Gut colonization by *K. pneumoniae* is increasingly recognized as a key risk factor for subsequent bloodstream infection.

The high levels of neonatal carriage found from initial samples on admission to the NICU may reflect high community prevalence. One study of rural Thai communities reported a prevalence of 69.3% for ESBL-positive carriage in stools [7]; another showed 29–51% ESBL-positive carriage in healthy people from three different rural areas of Thailand [6].

More than half of the patients were found to be carrying *Acinetobacter* spp. at some point during their NICU stay, with 30% becoming colonized after admission following initial negative samples. Over half were IPM-R. In contrast, in Cambodia, neonatal colonization with *Acinetobacter* spp. was rare, with 1.7% of patients becoming colonized after admission, and 6% of all *Acinetobacter* spp. positive samples were IPM-R [8]. Although most *Acinetobacter* spp. isolated in this study were from tracheal samples, over half of the patients with *Acinetobacter* spp. had positive results from their rectal swabs, with 70% IPM-R. High rates of healthcare-associated infection and colonization with *Acinetobacter* spp. have been reported previously in Thailand, especially in adult intensive care units, and some studies have found *Acinetobacter* spp. to be the most common hospital-acquired antibiotic-resistant organism [9], [10].

Nearly two-thirds of patients were colonized with carbapenem-resistant organisms in this study. Although the highest amount of carbapenem resistance was seen in *Acinetobacter* spp*.*, resistance was also common in *P. aeruginosa* and *K. pneumoniae.* In 2017, the World Health Organization classified carbapenem-resistant *A. baumannii, P. aeruginosa* and Enterobacteriaceae as pathogens of critical importance requiring research and development of new antimicrobials [1]. Carbapenem resistance in *A. baumannii* has previously been identified as a major problem in Thailand [11]. This may reflect high usage of carbapenems, which have increasingly become the treatment of choice as the prevalence of ESBL has risen.

A key limitation of this study was the lack of maternal carriage data, which prevents identification of the role of vertical transmission. Another limitation is that it was only possible to take initial samples from patients within 48 h of NICU admission in less than one-third of the patients. As a result, some of the patients with initial positive samples may have become positive between the time of admission and the initial sample being taken. Unfortunately, information on infection control practices and ward hygiene was not available. This information would be helpful for guiding future infection prevention. Also, lack of high-resolution genotyping data prevents reliable reconstruction of transmission pathways. This will be addressed in future work.

In conclusion, this study found high colonization and acquisition rates of antibiotic-resistant Gram-negative organisms from an NICU in Thailand. After day 13 of stay on the NICU, all patients that grew an organism were positive for an IPM-R organism, and after 22 days of NICU stay, all isolates were positive for an ESBL-positive organism. Once patients were found to be colonized by an ESBL-positive or IPM-R organism, they remained colonized for the rest of their NICU stay. Results from this study highlight the need to quantify the relative importance of selection and transmission, and develop effective interventions to reduce the resistance burden.

## Conflict of interest statement

None declared.

## Funding source

This study was supported by the UK Medical Research Council and Department for International Development (Grant No. MR/K006924/1 to BSC) and the Wellcome Trust as part of the Wellcome Trust-Mahidol University-Oxford Tropical Medicine Research Programme (Grant No. 106698/Z/14/Z).
