# Supplementary material for: Antimicrobial-resistant Gram-negative colonization in infants from a neonatal intensive care unit in Thailand
Source: J Hosp Infect. 2019 Oct;103(2):151–5. doi: 10.1016/j.jhin.2019.04.004 (PMC6863035; doi:10.1016/j.jhin.2019.04.004)
Supplement: Multimedia component 1 [file mmc1.docx]

**Supplementary material I**

**Materials and methods**

*Microbiological testing*

Colonization swabs were collected using sterile cotton-tipped swabs and Amies transport medium with charcoal (MWE Transwabs, Corsham, UK), and transported immediately to the microbiology laboratory where they were processed on the same day as collection. Swabs were inoculated on to MacConkey agar plates as well as KPC and ESBL agar (CHROMagar) to detect third-generation cephalosporin +/- carbapenem-resistant Gram-negative bacilli, including *Acinetobacter* spp., *Pseudomonas aeruginosa* and coliforms (*Escherichia coli*, *Enterobacter* spp. and *Klebsiella pneumoniae*). These organisms were chosen as they have been recognized as significant nosocomial pathogens/colonizers in the published literature. Cefpodoxime and imipenem discs (Oxoid, Basingstoke, UK) were added to MacConkey plates before overnight incubation in air at 37^o^C. Resistant organisms were identified by standard microbiological techniques [growth characteristics, Gram stain, biochemical profiles – triple sugar iron agar, urea agar, citrate agar, motility-indole-lysine agar (Oxoid/Becton Dickinson, Franklin Lakes, NJ, USA) and API 20E/20NE (bioMérieux, Marcy L’Etoile, France)]. Comprehensive antimicrobial resistance profiles were determined following current US Clinical and Laboratory Standards Institute (CLSI) guidelines (M100S, 26^th^ edition, January 2016) using disk diffusion on Mueller-Hinton agar (Oxoid). Extended-spectrum β-lactamase production was determined for *E. coli* and *K. pneumoniae* isolates using the double-disk method [cefotaxime +/- clavulanate and ceftazidime +/- clavulanate (Becton Dickinson)] following CLSI guidelines.

**Supplementary material II**

**Statistical analysis**

Univariable and multi-variable logistic regression models were used to determine factors associated with colonization with an extended-spectrum β-lactamase (ESBL)-positive or imipenem-resistant (IPM-R) organism from initial sampling for each patient. Variables were selected based on biological plausibility and substantive knowledge. Survival analysis using univariable and multi-variable Cox proportional hazard models was used to investigate factors associated with time to acquisition of an ESBL-positive or IPM-R organism during the first admission to the neonatal intensive care unit (NICU) amongst patients with an initial negative swab or stool result. Cumulative hazard functions for ESBL and IPM-R acquisition were obtained using the Nelson-Aalen estimator. Model fit of the logistic regression was assessed using Hosmer-Lemeshow test and examination of log-log plots, and scaled Schoenfeld residuals were used to assess the proportional hazards assumption in the Cox models. A Fine and Gray competing risk survival regression model was used to determine factors associated with the cumulative incidence of ESBL and IPM-R carriage acquisition accounting for the competing event of NICU discharge. These effect estimates are presented as subdistribution hazard ratios. Observations were censored at time of discharge or death.

A


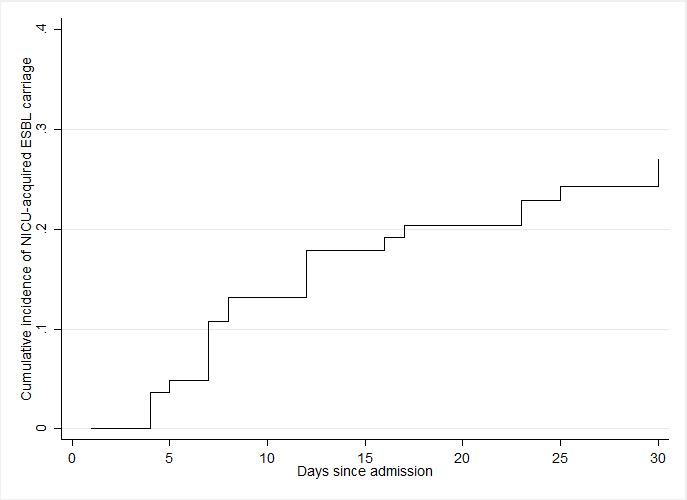

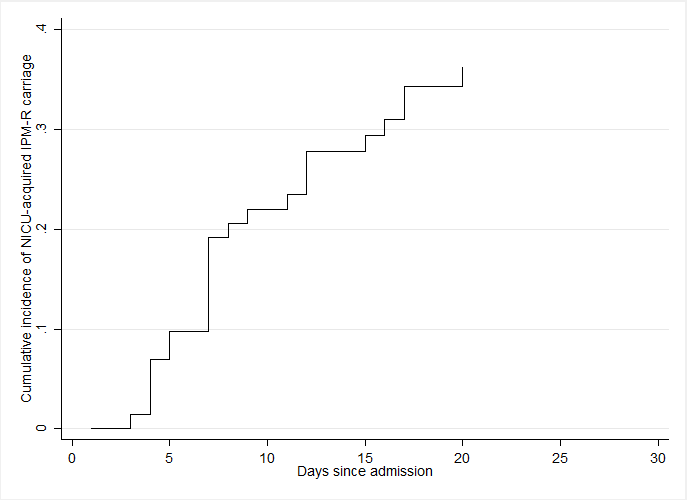


No. at risk

71 38 18 10 6 3 1

No. at risk

60 26 9 4 1

B

**Figure A.** (A) Cumulative incidence function for extended-spectrum beta-lactamase (ESBL) colonization in 71 patients admitted to the neonatal intensive care unit (NICU) and found to be non-colonized at admission. (B) Cumulative incidence function for colonization with an imipenem-resistant (IPM-R) organism in 60 infants admitted to the NICU and found to be non-colonized at admission.

**Table A**

Organism isolated and site of colonization from 660 samples from 97 infants admitted to the neonatal intensive care unit

| Organism | Rectum | Stool | Trachea | Total |
| --- | --- | --- | --- | --- |
| *Pseudomonas aeruginosa* | 113 | 24 | 86 | 223 |
| *Acinetobacter baumanii* | 79 | 17 | 94 | 190 |
| *Acinetobacter lwoffii* | 0 | 0 | 2 | 2 |
| *Escherichia coli* | 36 | 2 | 0 | 38 |
| *Klebsiella pneumoniae* | 194 | 41 | 87 | 322 |
| *Enterobacter* spp. | 47 | 10 | 58 | 115 |
| No growth | 82 | 20 | 63 | 165 |
| Total | 551 | 114 | 390 | 1055 |

**Table B**

Number of organisms tested against antibiotics which were resistant (R) or susceptible (S)

| Organism (*N*) | Antibiotic and number of organisms tested against it which were R or S | | | | | | | | | | | | | | | | | | | |
| --- | --- | --- | --- | --- | --- | --- | --- | --- | --- | --- | --- | --- | --- | --- | --- | --- | --- | --- | --- | --- |
|  | AMP R | AMP S | AMC R | AMC S | CRO R | CRO S | CAZ R | CAZ S | CIP R | CIP S | CN R | CN S | SXT R | SXT S | C R | C S | IPM R | IPM S | CT R | CT S |
| *Pseudomonas aeruginosa* (223) | - | - | - | - | - | - | 209 | 12 | 79 | 142 | 79 | 141 | - | - | - | - | 220 | 1 | 3 | 218 |
| *Acinetobacter baumannii* (190) | - | - | - | - | 186 | 1 | 184 | 3 | 182 | 6 | 183 | 5 | 172 | 16 | - | - | 184 | 5 | - | - |
| *Acinetobacter lwoffii* (2) | - | - | - | - | 2 | 0 | 2 | 0 | 0 | 2 | 0 | 2 | 0 | 2 | - | - | 2 | 0 | - | - |
| *Escherichia coli* (38) | 36 | 0 | 8 | 29 | 33 | 4 | 30 | 7 | 4 | 33 | 28 | 9 | 20 | 17 | 24 | 14 | 7 | 31 | - | - |
| *Klebsiella pneumoniae* (322) | 322 | 0 | 302 | 19 | 316 | 5 | 312 | 8 | 300 | 20 | 309 | 12 | 304 | 17 | 25 | 297 | 273 | 48 | - | - |
| *Enterobacter* spp. (115) | 114 | 0 | 114 | 0 | 114 | 0 | 100 | 14 | 1 | 114 | 108 | 5 | 21 | 93 | 12 | 91 | 111 | 3 | - | - |

AMP, ampicillin; AMC, co-amoxiclav; CRO, ceftriaxone; CAZ, ceftazidime; CIP, ciprofloxacin; CN, gentamicin; SXT, co-trimoxazole; C, chloramphenicol; IPM, imipenem; CT, colistin.

**Table C**

Results of univariable logistic regression models for factors associated with colonization with an extended-spectrum beta-lactamase (ESBL)-positive *Escherichia coli* or *Klebsiella pneumoniae* at first admission to the neonatal intensive care unit (NICU), and colonization with an imipenem-resistant (IPM-R) *E. coli,* *K. pneumoniae*, *Acinetobacter baumannii* or *Pseudomonas aeruginosa* at first admission to the NICU (97 infants)

| Factor | Infants with factor  *N* (%) | Infants with factor with ESBL  *N* (%) | Univariable analysis ESBL from initial sample | | | Infants with factor with  IPM-R organism  *N* (%) | Univariable analysis IPM-R organism from initial sample | | |
| --- | --- | --- | --- | --- | --- | --- | --- | --- | --- |
|  |  |  | OR | 95% CI | *P*-value |  | OR | 95% CI | *P*-value |
| Premature | 66 (68) | 19 (29) | 1.61 | 0.60–4.33 | 0.341 | 27 (41) | 1.38 | 0.58–3.32 | 0.466 |
| Birth asphyxia | 25 (26) | 7 (24) | 1.05 | 0.38–2.89 | 0.925 | 13 (52) | 2.12 | 0.85–5.33 | 0.109 |
| Sex (male) | 49 (50) male,  48 (49) female | 19 (39) male,  8 (16) female | 3.06 | 1.19–7.92 | 0.021 | 26 (53) male,  11 (23) female | 3.95 | 1.65–9.45 | 0.002 |
| Ventilation- endotracheal tube | 80 (82) | 22 | 1.06 | 0.34–3.30 | 0.917 | 35 (44) | 4.15 | 1.12–15.34 | 0.033 |

OR, odds ratio; CI, confidence interval.

**Table D**

Results of multi-variable logistic regression models for factors associated with colonization with an extended-spectrum beta-lactamase (ESBL)-positive *Escherichia coli* or *Klebsiella pneumoniae* at first admission to the neonatal intensive care unit (NICU) and colonization with an imipenem-resistant (IPM-R) *E. coli,* *K. pneumoniae*, *Acinetobacter baumannii* or *Pseudomonas aeruginosa* at first admission to the NICU (97 infants)

| Factor | Infants with factor  *N* (%) | Infants with factor with ESBL  *N* (%) | Multi-variable analysis ESBL from initial sample | | | Infants with factor with  IPM-R  *N* (%) | Multi-variable analysis IPM-R organism from initial sample | | |
| --- | --- | --- | --- | --- | --- | --- | --- | --- | --- |
|  |  |  | OR | 95% CI | *P*-value |  | OR | 95% CI | *P*-value |
| Premature | 66 (68) | 19 (29) | 1.66 | 0.60–4.61 | 0.332 | 27 (41) | 1.47 | 0.57–3.81 | 0.422 |
| Birth asphyxia | 25 (26) | 7 (24) | 0.93 | 0.32–2.72 | 0.898 | 13 (52) | 1.79 | 0.66–4.83 | 0.251 |
| Sex (male) | 49 (50) male, 48 (49) female | 19 (39) male, 8 (16) female | 3.23 | 1.22–8.57 | 0.018 | 26 (53) male,  11 (23) female | 3.75 | 1.52–9.22 | 0.004 |
| Ventilation- endotracheal tube | 80 (82) | 22 | 0.82 | 0.25–2.72 | 0.744 | 35 (44) | 3.33 | 0.84–13.23 | 0.087 |

OR, odds ratio; CI, confidence interval.

**Table E**

Results of univariable and multi-variable Cox proportional hazards models to define factors affecting time to colonization by an extended-spectrum beta-lactamase (ESBL)-positive *Escherichia coli* or *Klebsiella pneumoniae* isolate in 71 infants admitted to the neonatal intensive care unit and found to be non-colonized at admission

| Factor | Infants with factor  *N* (%) | Infants with factor with ESBL  *N* (%) | HR for acquiring ESBL during admission | | | | | |
| --- | --- | --- | --- | --- | --- | --- | --- | --- |
|  |  |  | Univariable model | | | Multi-variable model | | |
|  |  |  | HR | 95% CI | *P*-value | HR | 95% CI | *P*-value |
| Premature | 46 (65) | 15 (33) | 0.32 | 0.11–0.88 | 0.027 | 0.31 | 0.11–0.88 | 0.028 |
| Birth asphyxia | 18 (25) | 11 (61) | 0.96 | 0.40–2.3 | 0.919 | 0.94 | 0.38–2.33 | 0.899 |
| Sex (male) | 71  31 (44) male,  40 (56) female | 11 (35) male,  13 (33) female | 0.57 | 0.24–1.38 | 0.212 | 0.59 | 0.24–1.43 | 0.243 |
| Ventilation- endotracheal tube | 58 (82) | 22 (38) | 0.91 | 0.12–7.17 | 0.931 | 0.65 | 0.01–5.45 | 0.695 |

HR, hazard ratio; CI, confidence interval.

**Table F**

Results of univariable and multi-variable Cox proportional hazards models to define factors affecting time to colonization by an imipenem-resistant (IPM-R) *Escherichia coli,* *Klebsiella pneumoniae*, *Acinetobacter baumannii* or *Pseudomonas aeruginosa* in 60 infants admitted to the neonatal intensive care unit and found to be non-colonized at admission

| Factor | Infants with factor  *N* (%) | Infants with factor and an IMP-R organism *N* (%) | HR for acquiring IPM-R organism during admission | | | | | |
| --- | --- | --- | --- | --- | --- | --- | --- | --- |
|  |  |  | Univariable model | | | Multi-variable model | | |
|  |  |  | HR | 95% CI | *P*-value | HR | 95% CI | *P*-value |
| Premature | 38 (63) | 15 (39) | 0.27 | 0.11–0.66 | 0.004 | 0.26 | 0.10–0.69 | 0.007 |
| Birth asphyxia | 12 (20) | 7 (58) | 1.63 | 0.66–4.06 | 0.292 | 1.29 | 0.46–3.57 | 0.630 |
| Sex (male) | 37 (62) male,  23 (38) female | 15 (41) male,  10 (43) female | 1.46 | 0.65–3.32 | 0.362 | 1.45 | 0.61–3.43 | 0.396 |
|  |  |  |  |  |  |  |  |  |
| Ventilation- breathing tube | 45 (75) | 22 (49) | 0.59 | 0.16–2.13 | 0.422 | 0.41 | 0.11–1.55 | 0.188 |

HR, hazard ratio; CI, confidence interval.

**Table G**

Results of univariable Fine and Gray competing risks regression model showing the subhazard ratio (SHR) to define factors affecting time to colonization by an extended-spectrum beta-lactamase (ESBL)-positive *Escherichia coli* or *Klebsiella pneumoniae* isolate in 71 infants, and colonization with an imipenem-resistant (IPM-R) *E. coli,* *K. pneumoniae*, *Acinetobacter baumannii* or *Pseudomonas aeruginosa* in 60 infants admitted to the neonatal intensive care unit and found to be non-colonized at admission

| Factor | Univariable analysis for acquiring an ESBL during admission | | | | | Univariable analysis for acquiring an IPM-R organism during admission | | | | |
| --- | --- | --- | --- | --- | --- | --- | --- | --- | --- | --- |
|  | Infants with factor  *N* (%) | Infants with factor with an ESBL  *N* (%) | SHR | 95% CI | *P*-value | Infants with factor  *N* (%) | Infants with factor with an IPM-R organism  *N* (%) | SHR | 95% CI | *P*-value |
| Premature | 46 (65) | 15 (33) | 0.93 | 0.39–2.22 | 0.879 | 38 (63) | 15 (39) | 0.72 | 0.32–1.6 | 0.417 |
| Birth asphyxia | 18 (25) | 11 (61) | 3.14 | 1.44–6.88 | 0.004 | 12 (20) | 7 (58) | 1.79 | 0.78–4.09 | 0.167 |
| Sex (male) | 31 (44) male,  40 (62) female | 10 (32) male,  13 (33) female | 0.98 | 0.44–2.21 | 0.44 | 37 (61) male,  23 (38) female | 15 (41) male,  10 (43) female | 1.14 | 0.52–2.5 | 0.734 |
| Ventilation- endotracheal tube | 58 (82) | 22 (38) | 5.69 | 0.74–44.07 | 0.096 | 45 (75) | 22 (49) | 2.73 | 0.78–9.56 | 0.177 |

**Table H**

Results of multi-variable Fine and Gray competing risks regression model showing the subhazard ratio (SHR) to define factors affecting time to colonization by an extended-spectrum beta-lactamase (ESBL)-positive *Escherichia coli* or *Klebsiella pneumoniae* isolate in 71 infants, and colonization with an imipenem-resistant (IPM-R) *E. coli,* *K. pneumoniae*, *Acinetobacter baumannii* or *Pseudomonas aeruginosa* in 60 infants admitted to the neonatal intensive care unit and found to be non-colonized at admission

| Factor | Infants with factor  *N* (%) | Infants with factor with an ESBL  *N* (%) | Multi-variable analysis for acquiring an ESBL during admission | | | Infants with factor  *N* (%) | Infants with factor with an IPM-R organism  *N* (%) | Multi-variable analysis for acquiring an IPM-R organism during admission | | |
| --- | --- | --- | --- | --- | --- | --- | --- | --- | --- | --- |
|  |  |  | SHR | 95% CI | *P*-value |  |  | SHR | 95% CI | *P*-value |
| Premature | 46 (65) | 15 (33) | 0.94 | 0.42–2.12 | 0.877 | 38 (63) | 15 (39) | 0.73 | 0.32–1.64 | 0.440 |
| Birth asphyxia | 18 (25) | 11 (61) | 2.71 | 1.25–5.87 | 0.011 | 12 (20) | 7 (58) | 1.45 | 0.62–3.38 | 0.394 |
| Sex (male) | 31 (44) male,  40 (62) female | 10 (32) male,  13 (33) female | 1.07 | 0.49–2.32 | 0.869 | 37 (61) male,  23 (38) female | 15 (41) male,  10 (43) female | 0.98 | 0.45–2.18 |  |
|  |  |  |  |  |  |  |  |  |  | 0.969 |
| Ventilation- endotracheal tube | 58 (82) | 22 (38) | 4.52 | 0.56–36.39 | 0.156 | 45 (75) | 22 (49) | 2.67 | 0.72–9.94 | 0.144 |
